# *Leishmania* Parasites Drive PD-L1 Expression in Mice and Human Neutrophils With Suppressor Capacity

**DOI:** 10.3389/fimmu.2021.598943

**Published:** 2021-06-15

**Authors:** Alessandra M. da Fonseca-Martins, Phillipe de Souza Lima-Gomes, Maísa Mota Antunes, Renan Garcia de Moura, Luciana P. Covre, Carolina Calôba, Vivian Grizente Rocha, Renata M. Pereira, Gustavo Batista Menezes, Daniel Claudio Oliveira Gomes, Elvira M. Saraiva, Herbert L. de Matos Guedes

**Affiliations:** ^1^ Laboratório de Imunofarmacologia, Instituto de Biofísica Carlos Chagas Filho, Universidade Federal do Rio de Janeiro, Rio de Janeiro, Brazil; ^2^ Departamento de Imunologia, Laboratório de Imunobiologia das Leishmanioses, Instituto de Microbiologia Paulo de Góes, Universidade Federal do Rio de Janeiro, Rio de Janeiro, Brazil; ^3^ Departamento de Imunologia, Laboratório de Imunobiotecnologia, Instituto de Microbiologia Paulo de Góes, Universidade Federal do Rio de Janeiro, Rio de Janeiro, Brazil; ^4^ Center for Gastrointestinal Biology, Departamento de Morfologia, Instituto de Ciências Biológicas, Universidade Federal de Minas Gerais, Minas Gerais, Brazil; ^5^ Núcleo de Doenças Infecciosas, Universidade Federal do Espírito Santo, Vitória, Brazil; ^6^ Division of Medicine, University College London, London, United Kingdom; ^7^ Departamento de Imunologia, Laboratório de Imunologia Molecular, Instituto de Microbiologia Paulo de Góes, Universidade Federal do Rio de Janeiro, Rio de Janeiro, Brazil; ^8^ Laboratório Interdisciplinar de Pesquisas Médicas, Instituto Oswaldo Cruz, Fundação Oswaldo Cruz, Rio de Janeiro, Brazil

**Keywords:** PD-L1, neutrophils, skin, *Leishmania*, human cutaneous leishmaniasis, murine leishmaniasis, supression

## Abstract

Neutrophils play an important role in the outcome of leishmaniasis, contributing either to exacerbating or controlling the progression of infection, a dual effect whose underlying mechanisms are not clear. We recently reported that CD4^+^ and CD8^+^ T cells, and dendritic cells of *Leishmania amazonensis-*infected mice present high expression of PD-1 and PD-L1, respectively. Given that the PD-1/PD-L1 interaction may promote cellular dysfunction, and that neutrophils could interact with T cells during infection, we investigated here the levels of PD-L1 in neutrophils exposed to *Leishmania* parasites. We found that both, promastigotes and amastigotes of *L. amazonensis* induced the expression of PD-L1 in the human and murine neutrophils that internalized these parasites *in vitro*. PD-L1-expressing neutrophils were also observed in the ear lesions and the draining lymph nodes of *L. amazonensis*-infected mice, assessed through cell cytometry and intravital microscopy. Moreover, expression of PD-L1 progressively increased in neutrophils from ear lesions as the disease evolved to the chronic phase. Co-culture of infected neutrophils with *in vitro* activated CD8^+^ T cells inhibits IFN-γ production by a mechanism dependent on PD-1 and PD-L1. Importantly, we demonstrated that *in vitro* infection of human neutrophils by *L braziliensis* induced PD-L1^+^ expression and also PD-L1^+^ neutrophils were detected in the lesions of patients with cutaneous leishmaniasis. Taken together, these findings suggest that the *Leishmania* parasite increases the expression of PD-L1 in neutrophils with suppressor capacity, which could favor the parasite survival through impairing the immune response.

## Importance

Neutrophils outnumber leukocytes in healthy human blood, rapidly migrate to infected or inflamed sites and have powerful mechanisms to eliminate pathogens. These cells secrete cytokines and chemokines critical for the initiation and amplification of the inflammatory response. Neutrophils also have the ability to modulate adaptive immune cells through a variety of receptors, including the programmed death ligand-1 (PD-L1), a cell surface protein that suppresses T cell activation. We report that *Leishmania amazonensis*, which can cause cutaneous leishmaniasis, severe anergic diffuse cutaneous and even visceral leishmaniasis, upon interaction with murine and human neutrophils induces the expression of PD-L1. Neutrophils expressing PD-L1 were observed in the ear lesions and draining lymph nodes of infected mice and in human cutaneous leishmaniasis biopsies. Our findings suggest that *Leishmania* could modulate PD-L1 expression in neutrophils, weakening the immune response to favor its survival, which opens up new possibilities of targeting PD-L1 for therapy.

## Introduction

Neutrophils, the most abundant white blood cells in the human circulation, play a crucial role in eliminating pathogens by multiple mechanisms, and participating in the development of the inflammatory reaction. Thus, neutrophils are key components of the innate immune response and in the elimination of infectious agents. They are recognized for their broad defense repertoire that includes the production of reactive oxygen species, phagocytosis, degranulation and the release of the antimicrobial neutrophil extracellular traps (1, 2).

Within the inflammatory environment, neutrophils interact with other immune cells, and secrete cytokines and chemokines critical for the development and establishment of the necessary conditions required for the interface with the adaptive immune response (3, 4). On the other hand, these cells can also favor the progression of various diseases, such as rheumatoid arthritis, systemic lupus erythematosus, and cystic fibrosis, as well as sepsis, HIV-1 infection, and malaria (5–8).

Neutrophils may also present opposing roles in infections caused by *Leishmania* sp. In fact, it has been shown that neutrophils can both exacerbate the disease or protect the host, an outcome that is dependent on a fine balance pertaining to the *Leishmania* species and host genetic background (9).

Furthermore, neutrophils are endowed with the ability to induce T cell suppression (10). Analyses of patients with visceral leishmaniasis (VL) showed an increased frequency of CD15^+^ neutrophils expressing high levels of arginase, an enzyme associated with immunosuppression, which, after successful treatment, returned to basal levels (11). In addition, co-culture of low-density HLA-DR^+^-neutrophils and lymphocytes, both from VL patients, with *Leishmania* antigen increased the expression of programmed death ligand-1 (PD-L1, also called B7-H1 and CD274) in neutrophils, and of the programmed death receptor 1 (PD-1, also called CD279) in lymphocytes. These findings led to the hypothesis that low-density HLA-DR^+^ neutrophils may be involved in promoting T-cell exhaustion (12).

PD-1 is a receptor found in Natural Killer cells, T cells, B cells and activated monocytes (13–15), while the PD-L1 ligand can be found in neutrophils, B cells, dendritic cells, macrophages, mesenchymal stem cells and in non-hematopoietic cells, such as epithelial cells, muscle cells and hepatocytes (16, 17). The function of this pathway has been widely studied revealing a dichotomous activity depending on the model in which it is applied, where often it can inhibit T cell proliferation and cytokine production or increase T cell activation (18, 19). Thus, the formation of the PD-1/PD-L1 complex can be seen as a mechanism used by pathogens to evade the immune response or as a possible regulator of tissue damage mediated by the immune system (20–22). The dysfunctional state characterized by increased expression of inhibitory receptors, like the PD-1/PD-L1 complex, and by progressive loss of function, is commonly known as cellular exhaustion (23).

The use of immunotherapy to reverse dysfunction due to T-cell exhaustion has been used successfully to treat several tumors (24, 25) and also in viral and parasitic infections (26, 27). Concerning leishmaniasis, we recently reported that mice infected with *L. amazonensis* presented high expression of PD-1 in CD4^+^ and CD8^+^ T cells, and PD-L1 in dendritic cells, and that treatment with anti-PD-1 or anti-PD-L1 antibodies reduced the parasite load and increased IFN-gamma production by CD4^+^ and CD8^+^ T cells, thus, reversing cell suppression (28).

It was demonstrated that neutrophils depletion using 1A8 increased T cells response against *Leishmania* (29). Here, we aimed to investigate whether the PD-L1 expression in murine and human neutrophils could be modulated after *in vitro* interaction with *Leishmania*, and identified PD-L1-expressing neutrophils in murine and human cutaneous lesions. We also evaluated *in vitro* the participation of PD-L1 expression by neutrophils in suppressing IFN-γ production by T cells.

## Materials And Methods

### Experimental Animals

Female BALB/c mice and C57BL/6, 6-8 weeks old, from the Central Bioterium (Centro de Ciências da Saúde – Universidade Federal do Rio de Janeiro, Brazil), were housed in ventilated mini-isolators (Alesco, Brazil) and kept under controlled temperature and light conditions. All animal experiments were performed in strict accordance with the Brazilian animal protection law (Lei Arouca, Number: 11.794/08) of the National Council for the Control of Animal Experimentation (CONCEA, Brazil). The protocol was approved by the Committee for Animal Use of the Universidade Federal do Rio de Janeiro (Permit Number: 161/18).

### Parasite Culture

For *in vivo* infection, *L. amazonensis* promastigotes (MHOM/BR/75/Josefa) were used. Parasites were first obtained from the macerated lesions of infected BALB/c mice and then grown at 26°C in M-199 medium (Cultilab) with 20% heat inactivated fetal bovine serum (FBS, Cultilab). Parasites were used for the experimental infections until the fifth passage in culture.

For *in vitro* infection, *L. amazonensis* promastigotes (RAT/BA/74/LV78) and *L. braziliensis* (MHOM/BR/2005/RPL5) were cultured at 26°C in Schneider medium (Invitrogen) with 20% FBS and 50 µg/mL gentamicin (Sigma). The parasites were used in assays until the fifth passage.

Amastigotes of *L. amazonensis* (RAT/BA/74/LV78) and *L. braziliensis* (MHOM/BR/2005/RPL5) were obtained from a culture of promastigotes maintained at 32°C in Grace’s medium (Invitrogen), pH 5.3 supplemented with 20% FBS and 25 µg/ml gentamicin. The amastigotes were used until the third passage in culture.

### Recruitment and Isolation of Murine Peritoneal Neutrophils

Neutrophils were recruited in BALB/c or C57BL/6 mice 3 h after intraperitoneal injection of 1 ml of 9% casein solution (Sigma). Mice were euthanized and the peritoneal cavity was washed with RPMI 1640 (Sigma) without serum at room temperature. A fraction of the obtained cells was first characterized by flow cytometry using anti-Ly6G-PerCP (murine, eBioscience) revealing >95% Ly6G^+^ cells (neutrophils). Remaining cells were centrifuged at 400 g for 5 min, resuspended in RPMI and used in the following assays.

### Purification of Murine Neutrophils From Bone Marrow

Bone marrow cells were flushed from the femur and tibia of BALB/c mice with RPMI/10% FBS into a 15 ml conical tube through a 100 μm cell strainer, and then were centrifuged at 400 g for 7 min at 24°C. The cell pellet was resuspended in RPMI/10% FBS and centrifuged at 1500 g for 30 min at 24°C in a Percoll gradient (100%, 72%, 65% and 58% v/v GE Healthcare). Neutrophils collected at the interface of 65% and 72%, were washed with PBS at 400 g for 10 min at 15°C, resuspended in RPMI and used in the following assays. Cell yield was ≥80% Ly6G^+^ cells (neutrophils) as analyzed by flow cytometry using anti-Ly6G-PerCP (murine, eBioscience).

### Isolation of Dermal Cells From Mouse Ears

Mice were infected intradermally in the right ear with 2x10^6^ stationary-phase promastigotes of *L. amazonensis* in 20 µl PBS. After 18 h, 15 days and 60 days post-infection, the ears were collected. Control was performed with ears from naïve mice. The dermal sheets were opened and added to the wells of a 24-well plate in DMEM (Sigma) with 1% penicillin/streptomycin and 100 µg/ml each of collagenase I and II (Sigma), followed by 90 min incubation at 37°C. The tissue was then macerated with a tissue mixer for 3 min, filtered through a 70 µm filter and washed with PBS at 400 g for 5 min at 15°C. Cells were resuspended in RPMI/10% FBS for further use.

### Isolation of Cells From the Lymph Nodes Draining the Infected Lesion

Mice were infected intradermally in the right footpad with 2x10^6^ *L. amazonensis* stationary-phase promastigotes in 20 µl PBS. After approximately 60 days post-infection, draining lymph nodes were removed, individually macerated and the cell suspensions were centrifuged at 400 g for 5 min at 4°C. The cell pellet was resuspended in 2 ml RPMI/10% FBS for further use.

### Purification of Human Blood Neutrophils

Human neutrophils from healthy blood were obtained by centrifugation on Ficoll Histopaque density gradient (Sigma-Aldrich), followed by hypotonic lysis (155 mM NH_4_Cl, 10 mM KHCO_3_, 0.1 mM EDTA, pH 7.4) to remove red blood cells. Isolated neutrophils (≥95% of the cells) were washed in PBS and resuspended in RPMI. All the procedures dealing with human blood were performed in accordance with the guidelines of the Research Ethics Committee (Hospital Universitário Fraga Filho, UFRJ, Brazil), protocol number: 4261 015400005257.

### *In Vivo* Infection

BALB/c mice were infected with 2x10^6^ *L. amazonensis* stationary-phase promastigotes in 20 µl PBS, either intradermally in the right ear or subcutaneously in the right footpad. The lesions were followed weekly for around two months by measuring the thickness with a pachymeter.

### *Leishmania*-Neutrophil Interaction *In Vitro*

Parasites were washed twice with PBS and incubated with 0.5 µM CFSE (carboxyfluorescein succinimidyl ester - Invitrogen) at 37°C. After 15 min, RPMI/20% FBS was added, and parasites incubated for a further 15 min on ice, followed by three washes with PBS, and resuspension in PBS. Murine neutrophils were infected with CFSE-labeled promastigotes or amastigotes of *L. amazonensis* (cell:parasite-MOI 1: 10) for 4 h at 35°C and human neutrophils were infected with CFSE-labeled promastigotes or amastigotes of *L. amazonensis* or *L. braziliensis* (MOI 1:5 or 1:10) for 4 h at 35°C.

### Cell Staining for Flow Cytometry

Cells from lymph nodes (5x10^5^) or ear homogenates (1x10^6^) were washed with PBS at 400 g for 5 min at 4°C and blocked with Human FcX (BioLegend) for 15 min, followed by staining with the antibody cocktail for 30 min at 4°C. Cells were then washed with a cytometry buffer (PBS with 5% FBS) at 400 g for 5 min and 4°C), then fixed with 4% formaldehyde (Sigma) for 15 min at 4°C. Cells were washed and resuspended in the cytometry buffer and stored in the dark at 4°C until acquisition. The following antibodies were used: anti-PD-L1-APC, anti-CD10-APC-780 (human, eBioscience); anti-CD45-APCcy7, anti-CD11b-FITC, anti-CD11b-PE, anti-CD11b-Pecy7, anti-Ly6G-PerCP, anti-Ly6G-FITC and anti-PD-L1-APC (murine, eBioscience). Acquisition of events (lymph node, 100,000 events; ear, all cells) was performed on a BD FACSAria™. The gate strategy was performed based on the selection of cell size (FSC) and composition (SSC). After identifying the main population, a gate of FSC-A (area) and FSC-H (weight) was used, where cellular doublets were excluded. Gates for positive events were established through Fluorescence Minus One (FMO) control. The data analyzes were performed using the FlowJo software.

### Imaging PD-L1 Expression by Intravital Microscopy

*L. amazonensis* ear-infected BALB/c mice (naïve-not infected; control with saline; 1 h, 7 days and 15 days and 60 days post-infection) were injected on the ear with 8-12 µl/animal of anti-PD-L1-APC and Ly6G-FITC antibodies. After 2 h, mice were anesthetized with ketamine and xylazine intraperitoneally. Images were obtained using a Nikon Eclipse Ti with an A1R confocal head equipped with four different lasers (excitation: 405, 488, 546 and 647 nm) and emission bandpass filters at 450/50, 515/30, 584/50 and 663/738 nm. Objective Plan Apo λ 10x. Analysis was performed using Volocity 6.3 software (PerkinElmer).

### CD8^+^ T Cells Activation And Culture *In Vitro*

Spleens were harvested from 6 to 8-week-old mice. Naïve CD8^+^ T cells (CD44^lo^ CD62L^hi^) T cells were purified (>95% purity) by negative selection (Dynabeads™ Untouched™ Mouse CD8 Cells Kit, Invitrogen) from RBC-lysed single-cell suspensions from spleen followed by cell sorting. For stimulation, purified CD8^+^ T cells were plated at 10^6^ cells/ml in 24-well plates precoated with 0,3 mg/mL goat anti-hamster and coated with anti-CD3 (clone 2C11) and anti-CD28 (clone 37.51) (1 μg/mL), as previously described (30, 31). After, 48 h, cells were removed from the TCR signal and re-cultured by diluting 1:2 in media (DMEM (11995-065, Gibco) supplemented with 10% FBS (26140079, Gibco), 1x Penicillin/Streptomycin (15140-122, Gibco), 1x L-Glutamine (25030-081, Gibco), 1x MEM Vitamins (11120-052, Gibco), 1x Sodium Pyruvate (11360-070, Gibco), 1x Essential Amino Acids (M5550, Sigma),1x Non-Essential Amino Acids (11140-050, Gibco), 10 mM HEPES (15630-080, Gibco) and 50 μM β-Mercaptoethanol (M3148, Sigma), containing 200 U/ml final concentration of recombinant murine IL-2 (Peprotech). Every 24 h, cells were diluted 1:2 with fresh media containing IL-2. On day 5, cells were stained with anti-CD25-PE, anti-CD44-FITC, anti-CD62L-APC and anti-CD127-PEcy7 antibodies (Biolegend) for phenotypic analysis and were assessed by flow cytometry.

### Coculture of Murine Neutrophils and Activated CD8^+^ T Cells

C57BL/6 neutrophils (5x10^5^) recruited to the peritoneum with casein were infected with promastigotes of *L. amazonensis* (cell:parasite-MOI 1:5) by 4h. After that, we added 5 µg/ml anti-PD-1 (CD279, clone RMP1-14, catalog # BE0146, Bioxcell) or anti-PD-L1 (BMS-936559, Bristol-Myers Squibb) or isotype (IgG2a, SouthernBiotech) for 15 min in the culture. Following, activated CD8^+^ T cells were added (5x10^5^) to the culture and the media was supplemented with 200 U/ml recombinant murine IL-2 (Peprotech) for 18h. The supernatants were collected and IFN-gamma quantified by specific ELISA using a standard protocol (Peprotech), detection limits are 12 - 3000 pg/ml.

### Skin Biopsies and Study Subjects

Punch biopsies (8 mm in diameter) from the border of skin lesions were obtained from 7 untreated patients with cutaneous leishmaniasis (CL) attended at the University Hospital (HUCAM) of Universidade Federal do Espírito Santo, Brazil. The diagnosis of CL was based on clinical criteria including differential diagnosis, skin lesion analysis and parasite identification by microscopy. In addition, all patients in this study tested positive for the PCR/restriction fragment length polymorphism of *L. braziliensis* and reported no prior infections or treatment. The CL group consisted of 5 males and 2 females with illness duration ranging from 30 to 120 days, lesion sizes ranging from 50–200 mm^2^ and 1.42± 0.53 lesion per patient. All patients involved in this study progressed to clinical cure after Meglumine antimoniate treatment. Control skin punch biopsy specimens from 12 healthy volunteers living in a non-endemic area with no history of leishmaniasis were also obtained. Biopsy specimens were frozen in OCT compound (Sakura). Sections of 6 µM were obtained longitudinally to expose all skin layers and were placed on poly-L-lysine-coated slides (Star Frost^®^). Tissues were then fixed in acetone and ethanol and stored at -80°C until use. All participants (patients and healthy volunteers) were seronegative for HIV, HBV and HCV infections, and had no history of chemotherapy, radiotherapy or treatment with immunosuppressive medications within the last 6 months. They provided written informed consent, and study procedures were performed in accordance with the principles of the Declaration of Helsinki. This study was registered with the HUCAM Ethical Committee under the number 735.274.

### Immunofluorescence Staining and Analysis of Human Tissues

Briefly, sections were hydrated with PBS, blocked with a 1% bovine serum albumin (BSA- Sigma) solution for 20 min and incubated with the following primary antibodies: anti-elastase (1:400; US1481001; Merk KGaA) or anti-PD-L1 (1:200; clone: ABM4E54; ab210931; Abcam) overnight at 4°C. Afterwards, slides were washed with PBS and incubated with goat anti-rabbit Alexa Fluor 488 (1:400; A11008) or goat anti-mouse Alexa Fluor 568 (1:400; A21124 or A21134) secondary antibodies (Thermo Fischer Scientific) for 1 h at room temperature. Slides were mounted with Fluoroshield Mounting Medium with DAPI (Abcam, ab104139) and analyzed by manually selecting regions of interest (ROI) using the Chromoplex Staining Detection system (Leica Biosystems). Cell and marker frequencies were defined according to the evaluated marker and cell nuclei of each ROI. Slides were imaged with a 20x objective (200× magnification) on a fluorescence microscope (Leica DMi8) with a 710 Metahead (Zeiss) by z-stack tile-scans and counted manually by using computer-assisted image analysis (National Institutes of Health Image Software ImageJ 1.52j; https://imagej.nih.gov/ij/).

## Data Analysis

Results are expressed as mean ± SEM with confidence level *p* ≤ 0.05. For lesion development analysis, a two-way ANOVA with Bonferroni post-test was used. For multiple comparisons, a one-way ANOVA followed by Tukey pairing was performed. Cell analyzes were performed using the paired Student’s *t* test or Mann-Whitney test (indicated p value in the graph and legend). Skin biopsies were analyzed using Student’s t test with Welch correction or Mann-Whitney test. Data analysis was performed using GraphPad Prism^®^ 8.00 software.

## Results

### PD-L1 Expression on Neutrophils Upon Interaction With *Leishmania*

**Figure 1:**
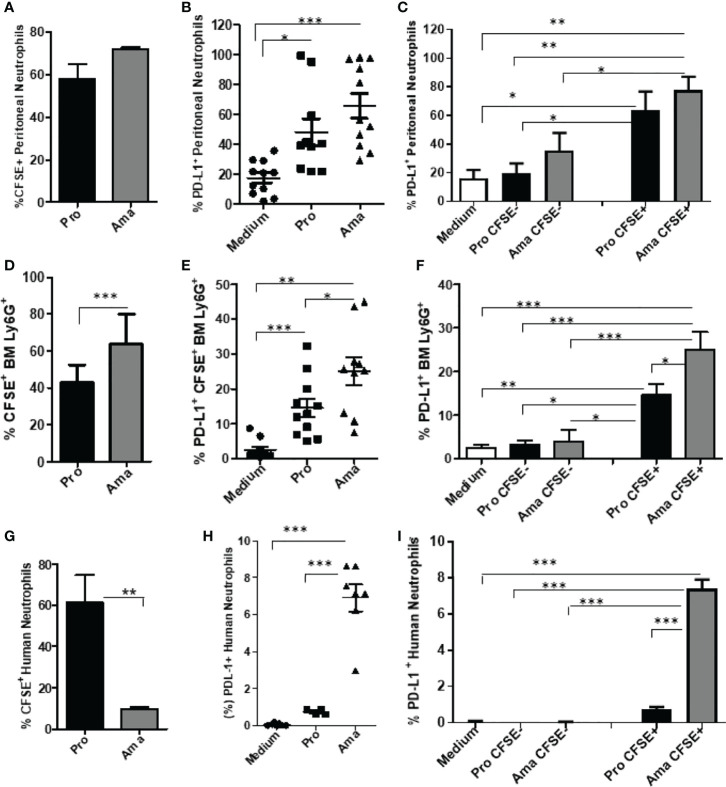
Increased expression of PD-L1 in murine and human neutrophils exposed to challenge with *L. amazonensis*. BALB/c neutrophils (5x10^5^) recruited to the peritoneum with casein, or purified from bone marrow, and human neutrophils (5x10^5^) from healthy donors were incubated with CFSE-stained promastigotes (Pro) and amastigotes (Ama) of *L. amazonensis* (murine 1:10; human 1:5), for 4 h. Control was performed with neutrophils on medium. Cells were then analyzed by flow cytometry using Ly6G^+^-Percp (neutrophils), CFSE^+^ (*L. amazonensis*) and PD-L1^+^-APC. **(A)** Percentage of peritoneal neutrophils infected with CFSE-labeled parasites. **(B)** Percentage of PD-L1^+^ peritoneal neutrophils upon interaction with parasites. **(C)** Percentage of PD-L1 expression on CFSE^-^ (bystander) and CFSE^+^ casein-recruited neutrophils. **(D)** Percentage of bone marrow (BM) neutrophils infected with CFSE-labeled parasites. **(E)** Percentage of PD-L1^+^ bone marrow neutrophils upon interaction with parasites. **(F)** Percentage of PD-L1 expression on CFSE^-^ and CFSE^+^ bone marrow neutrophils. **(G)** Percentage of human neutrophils infected with CFSE-labeled parasites. **(H)** Percentage of PD-L1^+^ human neutrophils upon interaction with parasites. **(I)** Percentage of PD-L1 expression on CFSE^-^ and CFSE^+^ human neutrophils. Data are mean ± SEM (N=10-11) and human donors (N=7). Each point represents a donor. *p < 0.01, **p < 0.001, ***p < 0.001.

Initially, we tested whether casein-recruited peritoneal neutrophils interacted equally with CSFE-labeled promastigotes and amastigotes of *L. amazonensis*. Our results evidenced that neutrophils internalized both parasite stages at similar levels (**Figure 1A**). Next, we analyzed the expression of PD-L1 on the CSFE^+^ neutrophils, which were the neutrophils that had internalized the parasites. We observed that uninfected neutrophils (medium) constitutively expressed PD-L1 at low levels, while both forms of the parasite induced PD-L1 expression in the infected casein-recruited peritoneal neutrophils (**Figure 1B**). It is important to note that within the neutrophil population, PD-L1 expression in cells that did not internalize the parasites (bystander CSFE^-^ neutrophils) was not augmented, expressing similar PD-L1 levels to the control (**Figure 1C**). Similarly, we evaluated the parasite interaction with neutrophils isolated from the bone marrow. Amastigotes were internalized significantly more than promastigotes by these neutrophils (**Figure 1D**). Again, internalization of both parasite forms significantly increased PD-L1 expression in relation to the uninfected control (**Figure 1E**). Interestingly, in the bone marrow neutrophils, amastigotes induced significantly more PD-L1 expression than promastigotes (**Figure 1E**). Like the casein-recruited neutrophils, constitutive PD-L1 expression of bystander bone marrow neutrophils, was not modulated by the presence of either form of the parasite (**Figure 1F**). Comparing the levels of PD-L1 expression of the peritoneal and bone marrow neutrophils, our results demonstrated that PD-L1 expression was higher in peritoneal neutrophils, independent of the parasite infection (**Figure S1**), suggesting that the inflamed milieu or the maturation stage of the neutrophils could influence PD-L1 expression.

We next tested the interaction of human neutrophils with promastigotes and amastigotes of *L. amazonensis* (MOI 1:5). Unlike mouse neutrophils, human neutrophils internalized promastigotes more than the amastigotes (**Figure 1G**). However, PD-L1 induction occurred similarly to murine neutrophils, being significantly more expressed after interaction with amastigotes (**Figure 1H**). Interestingly, the PD-L1 constitutive expression (medium) in human neutrophils (**Figure 1I**) was lower than in mouse peritoneal (**Figure 1C**) and bone marrow (**Figure 1F**) neutrophils. Surprisingly, a reduction in PD-L1 expression was observed after human neutrophil infection with promastigotes and amastigotes at a higher MOI, although maintaining the same pattern of higher PD-L1 expression induced by amastigotes in comparison to promastigotes (**Figure S2**). Together, these results suggest that PD-L1 expression in neutrophils is modulated upon parasite interaction.

### PD-L1 Expression in Cells From Ear Lesions of BALB/c Mice Infected With *L. amazonensis*

**Figure 2:**
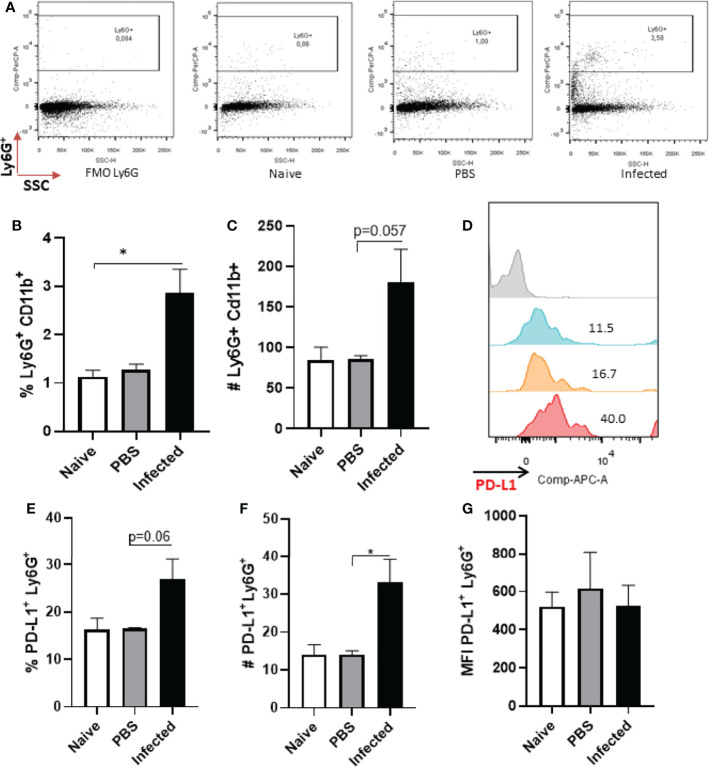
Analysis of murine ear neutrophils after 12h of infection. Cells were collected from *L. amazonensis*-infected ears after 12h then submitted to flow cytometry. Controls were performed with uninfected (naïve) and PBS injected mice. **(A)** Dot plot showing Ly6G^+^ cells (Ly6G-PerCP, SSC). **(B)** Percentage of Ly6G^+^ CD11b^+^ cells. **(C)** Number of Ly6G^+^ CD11b^+^ cells, p=0.057 (Mann-Whitney). **(D)** Histogram of PD-L1 expression. Grey = Fluorescence minus one control (FMO) PD-L1, Blue = Naïve, Orange = PBS and Red = Infected. **(E)** Percentage of PD-L1^+^ Ly6G^+^ cells, p=0,06 (Mann-Whitney). **(F)** Number of PD-L1^+^ Ly6G^+^ cells, p=0.06. **(G)** MFI of PD-L1^+^ Ly6G^+^. Data are mean ± SEM from cells of individual mice (N = 4-7 mice/group). *p < value 0,0323.

Following our observations of neutrophils *in vitro*, we assessed whether cells present at the lesion site *in vivo* would also express PD-L1 (**Figure S10**). As the peak of neutrophil infiltration has been reported to occur at 12 h after *Leishmania* infection in mice (29), we started by analyzing PD-L1 expression at this time-point. Our data shows increase in the percentage of Ly6G^+^ cells in infected group, but, while there is an increase in the number of neutrophils, it was not statistically different from the other groups (**Figures 2A–C**). Although the number of PD-L1^+^Ly6G^+^ increased in the infected group (**Figure 2F**), no differences were observed in the frequency of PD-L1^+^Ly6G^+^ or in the MFI between the groups (**Figures 2D, E, G**). Evaluating the same parameters after 18 h post infection, although the frequency of CD11b^+^ cells was a little higher in the infected ear, it was not significantly different from the naïve (non-infected) control; a similar observation was noted regarding the numbers of CD11b^+^ cells in naïve and infected mouse ears (**Figures S3A, B** and **S4A**). In addition, no differences were observed between the ears of naïve and infected mice in the frequency and number of Ly6G^+^CD11b^+^ cells (**Figures S3C, D** and **S4B**) and the expression of PD-L1 in the Ly6G^+^ cells (**Figures S3E–H**).

**Figure 3:**
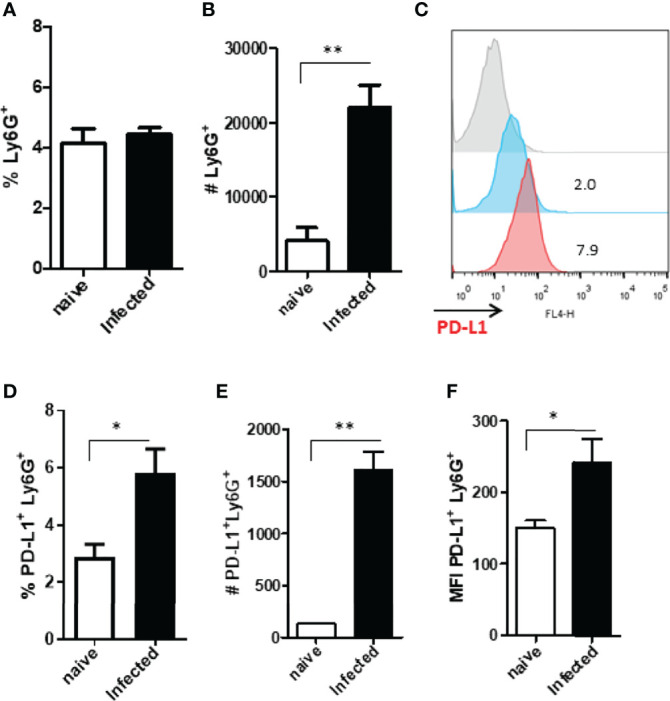
Analysis of murine ear neutrophils after 15 days of infection. Cells were collected from *L. amazonensis*-infected ears after 15 days then submitted to flow cytometry. Controls were performed with uninfected mice (naïve). **(A)** Percentage of Ly6G^+^ cells. **(B)** Number of Ly6G^+^ cells. **(C)** Histogram of PD-L1 expression. Grey = Fluorescence minus one control (FMO) PD-L1^+^, Blue = Naïve and Red = Infected. **(D)** Percentage of PD-L1^+^ Ly6G^+^ cells. **(E)** Number of PD-L1^+^ Ly6G^+^ cells. **(F)** MFI of PD-L1^+^ Ly6G^+^. Data are mean ± SEM from cells of individual mice (N = 4-5/group). *p < 0.03, **p < 0.005.

A second wave of neutrophils has been described to migrate to the infection site after 15 days (29), therefore we also evaluated PD-L1 expression at this time-point. Our results showed no statistical difference in the frequency of neutrophils between naïve and *Leishmania*-infected ear lesions (**Figure 3A**), however, a significant increase in the number of neutrophils was observed in infected mice compared with the naïve control (**Figure 3B**). Concomitantly, increased PD-L1 expression was detected in infected mice (**Figures 3C, D, F**) as well as an increased number of PD-L1-expressing neutrophils (**Figure 3E**).

**Figure 4:**
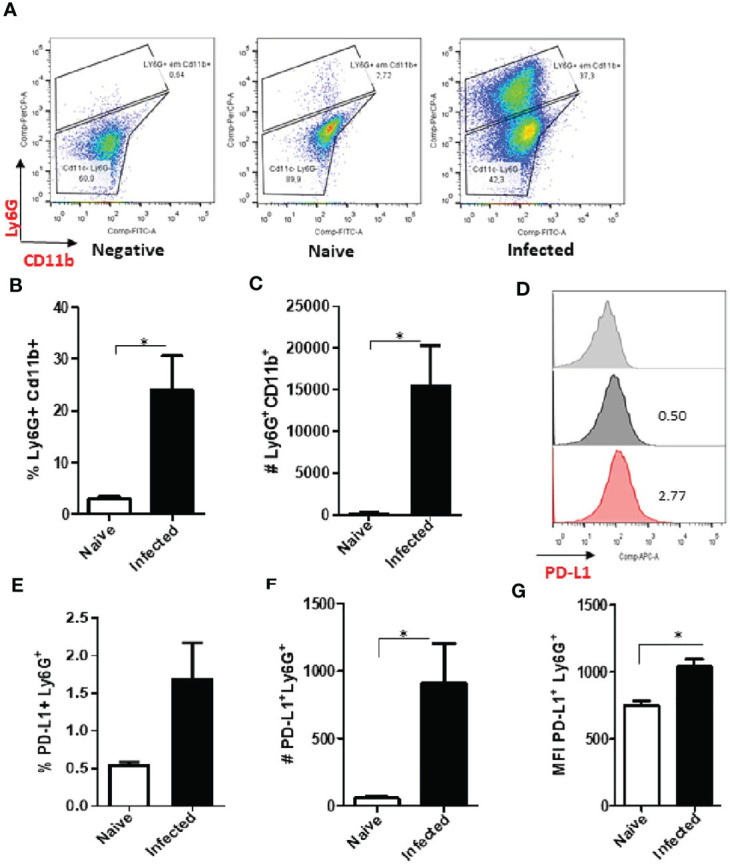
Increase of PD-L1^+^ Ly6G^+^ cells in *L. amazonensis*-infected ear lesions. Cells were collected from *L. amazonensis*-infected mouse ears after around 50 days. Controls were performed with uninfected mice (naïve). **(A)** Dot plot showing Ly6G^+^ CD11b^+^ cells (Ly6G-PerCP, CD11b-FITC). **(B)** Percentage of Ly6G^+^ CD11b^+^ cells. **(C)** Number of Ly6G^+^ CD11b^+^ cells. **(D)** Histogram of PD-L1 expression. Grey = Fluorescence minus one control (FMO) for PD-L1, Dark grey = Naïve and Red = Infected ear cells. **(E)** Percentage of PD-L1^+^ Ly6G^+^ cells, p=0.057. **(F)** Number of PD-L1^+^ Ly6G^+^ cells. **(G)** MFI of PD-L1^+^ Ly6G^+^. Data are mean ± SEM from two independent experiments (7-8 mice/group). *p < 0.02.

The analysis of cells from infected animals in the chronic phase, after 60 days of infection, displayed an increase in the neutrophil population (**Figures 4A–C** and **S5**). The expression of PD-L1 on neutrophils from 60-day lesions followed the same pattern with increased expression of PD-L1 in the cells from the infected animals (**Figures 4D–G**). These data show that as the lesion progresses, there is an increase in the population of neutrophils that express PD-L1, and this strengthens our hypothesis that a constant *Leishmania* stimulus promotes this induction.

### Intravital Images of Neutrophils Expressing PD-L1 in Infected Lesions but Not in Circulation

To validate our findings, we evaluated PD-L1 expression in neutrophils present in the lesions of *L. amazonensis*-infected mice directly *in vivo*. Initially, we analyzed the animals within 1 h of infection, since it is at this time that neutrophils begin the process of migrating to the infected site (32), in addition to the chronic lesion (60 days post-infection). Some migrating neutrophils in the tissue were expressing PD-L1, but interestingly, no expression was observed in the circulating neutrophils (**Figure S11**, **Movies S1**, **S2**). The absence of PD-L1 expression was observed in naïve and in saline-injected control mice (**Figures S11A, B**). After 1h post-infection, some migrating neutrophils in the tissue were expressing PD-L1, but interestingly, no expression was observed in the circulating neutrophils (**Figure S11C**; **Movies S1** and **S2**). Importantly, chronic lesions showed a high presence of neutrophils expressing PD-L1 (**Figure S11D**). In fact, the presence of PD-L1-expressing neutrophils, at times, formed a mass (**Movie S3**) that we could image only after cutting the lesion tissue. After this step, we could see the circulation contralateral to the injury (**Movie S4**). Interestingly, this contralateral circulation did not have circulating neutrophils expressing PD-L1. This observation is important because it demonstrates that the infection environment is conducive to the induction of the suppressive neutrophil without affecting the circulating neutrophils.

### PD-L1-Expressing Neutrophils in the Draining Lymph Nodes of Mice

**Figure 5:**
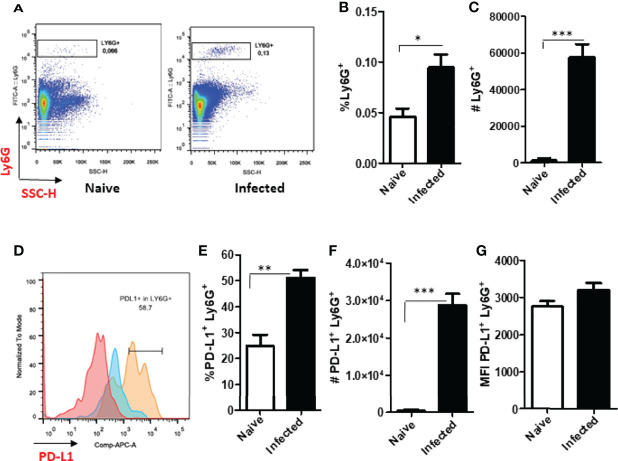
Increased expression of Ly6G^+^ and PD-L1^+^ in the draining lymph nodes of infected BALB/c mice. Mice were infected in the footpad with *L. amazonensis* promastigotes (2×10^6^). After approximately 60 days, the draining lymph nodes were collected, macerated and the cells analyzed by flow cytometry. Controls were performed with uninfected mice (naïve). **(A)** Dot plot showing Ly6G^+^ cells (Ly6G-FITC x SSC-H). **(B)** Percentage of Ly6G^+^ cells. **(C)** Number of Ly6G^+^ cells. **(D)** Histogram of PD-L1 (PD-L1-APC) expression. Red = Fluorescence minus one control (FMO) PD-L1; Blue = Naïve lymph nodes; Orange = Infected lymph nodes. **(E)** Percentage of PD-L1^+^ Ly6G^+^ cells. **(F)** Number of PD-L1^+^ Ly6G^+^ cells. **(G)** MFI of PD-L1^+^Ly6G^+^. Data are mean ± SEM of individual mice (5 mice/group) shown as a representative experiment of three producing the same result profile. *p < 0.01, **p < 0.001, ***p < 0.0001.

To better map PD-L1-expressing neutrophils we investigated these cells in the lymph nodes that drain the footpad lesions caused by *L. amazonensis*. Our results show that the neutrophil population was increased in the lymph nodes of the infected mice at 60 days (**Figures 5A–C**). The frequency and numbers of PD-L1-expressing neutrophils was also significantly increased (**Figures 5D–F**), however, the median intensity of fluorescence (MFI) was similar to the naïve control (**Figure 5G**). This data reveals that the neutrophils not only express PD-L1 at the infection site, but these cells, expressing the ligand, also have the ability to be drained to the lymph nodes.

### Neutrophils Expressing PD-L1 Suppress IFN-γ Production by T Cell

**Figure 6:**
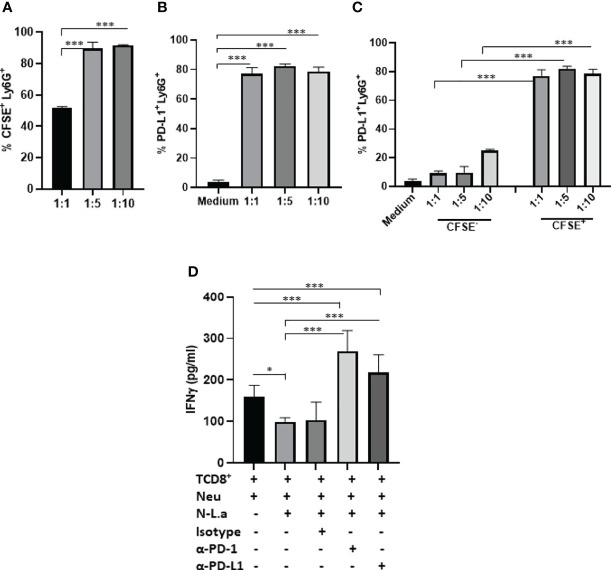
Coculture of murine neutrophils exposed to *L. amazonensis* with CD8^+^ T activated cells. C57BL/6 neutrophils (5x10^5^) recruited to the peritoneum with casein were incubated with CFSE-stained promastigotes (Pro) of *L. amazonensis* (1:1, 1:5, 1:10), for 4 h. Control performed with neutrophils on medium. Cells were then analyzed by flow cytometry using Ly6G^+^-Percp (neutrophils), CFSE^+^ (*L. amazonensis*) and PD-L1^+^-APC. **(A)** Percentage of CFSE^+^ Ly6G^+^ cells. **(B)** Percentage of PD-L1^+^ Ly6G+ cells. **(C)** Percentage of PD-L1 expression on CFSE^-^ (bystander) and CFSE^+^ cells. **(D)** Coculture of murine neutrophils (Neu) infected or not with promastigotes of *L. amazonensis* (Leish) with CD8^+^ T effectors cells. The coculture was treated or not with anti-PD-1 or anti-PD-L1 or isotype control for 18h. IFN-γ was analyzed in the culture supernatant by ELISA. Data are mean ± SEM. **(A–C)** N=3-4 and **(D)** N=7. *p=0.049, ***p < 0.001.

First, the expression of PD-L1 was evaluated in infected C57BL6 peritoneal neutrophils with *L. amazonensis* promastigotes (MOI 1:1; 1:5; 1:10). We observed 50% of infected neutrophils in 1:1 and 90% in 1:5 and 1:10 MOIs (**Figure 6A**). The expression of PD-L1 was not modulated by the infection rate, and similarly to the BALB/c neutrophils, bystander CSFE^-^ neutrophils expressed significantly less PD-L1 (**Figures 6B, C**). Following, the capacity of PD-L1^+^-neutrophils to inhibit the production of IFN-γ by CD8^+^ T was evaluated by co-culturing infected neutrophils with activated CD8^+^ T cells (**Figure 6D**). Characterization of isolated CD8^+^ T cells is shown in **Figure S9**. We observed a significant reduction in the IFN-γ production by CD8^+^ T cells co-cultured with infected neutrophil (1:5) compared to the non-infected neutrophils (Neu). Importantly, addition of anti-PD-L1 or anti-PD-1 to the co-culture restored the production of IFN-γ. Moreover, the isotype control was unable to reverse IFN-γ inhibition mediated by infected neutrophils. This result demonstrated that inhibition of IFN-γ production is dependent of PD-L1/PD-1 (**Figure 6D**).

### PD-L1-Expressing of Human Neutrophil *In Vitro* and in the Lesions Caused by *Leishmania braziliensis*

**Figure 7:**
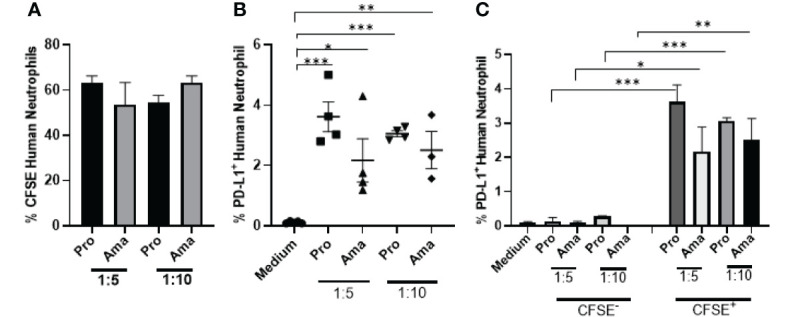
Increased expression of PD-L1 in human neutrophils exposed to *L. braziliensis*. Human neutrophils (5x10^5^) from healthy donors were incubated with CFSE-stained promastigotes (Pro) and amastigotes (Ama) of *L. braziliensis* (1:5 and 1:10), for 4 h. Control performed with neutrophils on medium. Cells were then analyzed by flow cytometry. **(A)** Percentage of neutrophils infected with CFSE-labeled parasites. **(B)** Percentage of PD-L1^+^ neutrophils upon interaction with parasites. **(C)** Percentage of PD-L1 expression on CFSE^-^ (bystander) and CFSE^+^ neutrophils. Data are mean ± SEM (N=4-5). *p < 0.0303, **p < 0.0047 ***p < 0.0001.

Finally, we evaluated the expression of PD-L1 in human neutrophils after *Leishmania braziliensis* infection. Differently from *L. amazonensis*, our results evidenced that independently of the MOI, neutrophils internalized both parasite stages of *L. braziliensis* at similar levels (**Figure 7A**). However, PD-L1 expression was higher in promastigotes infected-neutrophils and likewise *L. amazonensis*, PD-L1 was not expressed in the bystander neutrophils (**Figures 7B, C**).

**Figure 8:**
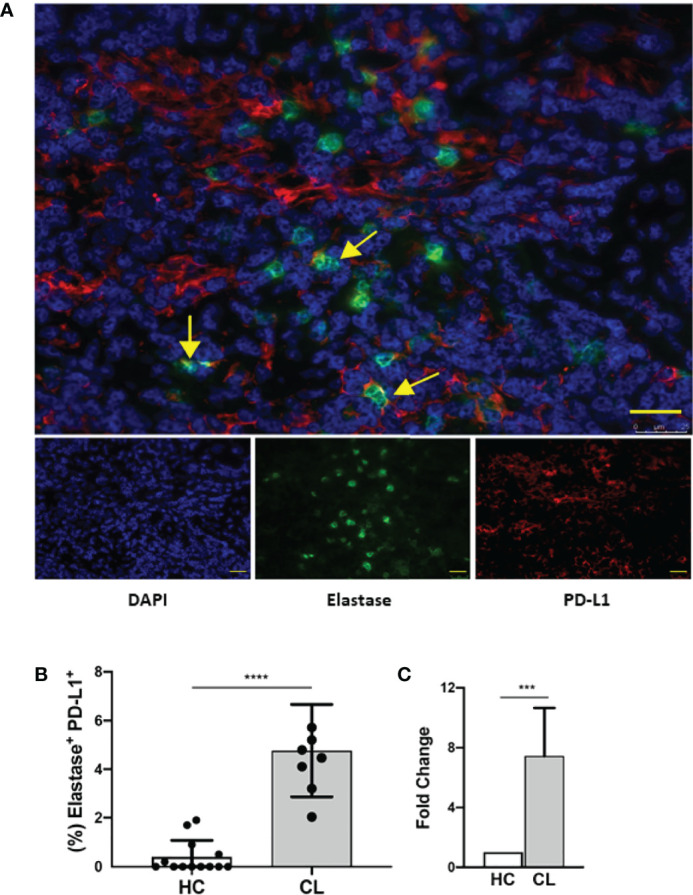
Human lesion neutrophils present a conspicuous expression of PD-L1. Healthy human skin and human cutaneous leishmaniasis skin lesions were stained with anti-elastase (green), anti-PD-L1 (red) and counter-stained with DAPI (blue). **(A)** Representative immunofluorescence staining of a cutaneous leishmaniasis lesion. Yellow arrows indicate anti-PD-L1 and anti-elastase double-stained cells. Bars = 50 µm. **(B)** Frequency of elastase-positive PD-L1^+^ cells of cumulative data of PD-L1 in neutrophils in healthy control skin (HC, N = 12) and in cutaneous leishmaniasis skin lesions (CL, N = 7). **(C)** Frequencies of double-stained neutrophils were normalized and data are expressed as fold over the control. Data shown as median with 95% confidence. The p values were calculated using Mann-Whitney test. ***p < 0.001.

We also investigated the PD-L1 expression on neutrophils from human cutaneous leishmaniasis biopsies obtained from untreated patients. It is already been demonstrated that cutaneous leishmaniasis lesions present hyperplasia with a dense inflammatory cellular infiltrate involving the dermal-epidermal junction, consisting mainly of lymphocytes, macrophages, neutrophils and plasma cells (33–38). Thus, we evaluated whether neutrophils in the lesions expressed PD-L1, which could directly impact on the skin immune balance during infection. Interestingly, lesional analysis revealed the expression of PD-L1 on neutrophils, that were respectively 7.44 and 3.77-fold higher than healthy controls (**Figures 8A–C** and **S6**), suggesting that neutrophils may have a role in human injuries caused by *L. braziliensis*, which still needs to be assessed.

## Discussion

In the present work we investigated the expression of PD-L1 in human and murine neutrophils, showing that upon interaction with promastigotes or amastigotes of *L. amazonensis*, these host cells express PD-L1, a molecule involved in T cell exhaustion. Interestingly, a higher number of murine bone marrow and human peripheral neutrophils expressing PD-L1 was evidenced after interaction with amastigotes than with promastigotes. Although, human neutrophils were less infected by *L. amazonensis* amastigotes than the murine neutrophils, this parasite stage induced higher numbers cells expressing PD-L1. However, these effects were not observed in casein-recruited murine neutrophils, where similar numbers of PD-L1^+^ neutrophils were stimulated by both parasite developmental stages, and which did not display any differences in neutrophil infection between the two parasite forms studied. A higher number of casein-recruited neutrophils expressed PD-L1 than bone marrow neutrophils upon interaction with both parasite stages. This result could be due to the inflammation induced by casein, since it has been shown that inflammation could contribute to a higher PD-L1 expression (16). The same lack of inflammatory stimuli could explain the low frequency of human PD-L1^+^ neutrophils in comparison with the murine neutrophils, but it should be noted that the frequency of PD-L1-expressing-neutrophils increased 8 times in relation to control (medium) after only 4h of interaction with the amastigotes. This high expression of PD-L1 in amastigote-infected neutrophils may be the consequence of its increased survival, consistent with the study highlighting the resistance of *L. amazonensis* amastigotes against microbicidal mechanisms of neutrophils, such as the oxidative burst (39).

Regardless of the source of neutrophils, the expression of PD-L1 was not affected in bystander cells irrespective of the presence of parasite forms in the co-culture. Taken together, this suggests that upon infection of neutrophils, the parasite induces this suppression marker in the host cell. Expression of PD-L1 has been described in monocytes from a patient presenting diffuse cutaneous leishmaniasis (40), and only in low-density neutrophils (normal-density neutrophils did not expressed PD-L1) from human visceral leishmaniasis patients (12), but, to the best of the authors’ knowledge, this is the first description of PD-L1 expression upon *in vitro* interaction with *Leishmania*. Interestingly, similar to our results, it was shown that incubation of neutrophils *ex vivo* with LPS for 6 h also induced PD-L1 expression (41).

A swarm of neutrophils are recruited to the *Leishmania* infection site and the presence of these cells has been reported in chronic lesions as well (32, 42–45). Thus, we investigated PD-L1 expression in neutrophils from a *L. amazonensis* cutaneous lesion in mouse ears at both these early and late time-points. Remarkably, although PD-L1^+^ neutrophils could already be observed after 12h or 18 h post-infection, and a significant number of PD-L1^+^ neutrophils was detected after 15 days post-infection, the number and frequency of which decreased a little in 2-month chronic lesions. However, the mean fluorescence intensity greatly enhanced (around 4 times) in the chronic lesion, compared to the lesion at 15 days. It was recently demonstrated that in the beginning of the infection (1h-24h) (46), resident macrophages are the predominant infected cells. In the absence of infection, neutrophils are not stimulated to express PD-L1 as observed in the frequency in 12 or 18 hours post *in vivo* infection. In the chronic phase the combination of infected neutrophils and the inflammatory cytokines can prime the express of PD-L1 as observed in 15, 30 and 60 days post infection.

Next, we sought to image the environment of an *L. amazonensis* infection *in vivo*, and our results showed that as the infection progressed, there was an accumulation of neutrophils at the site of infection, and some of them expressing PD-L1. The presence of neutrophils is in agreement with reports describing these cells in chronic lesions (60 days post infection) of animals naturally or experimentally infected with *Leishmania* (42–44). Importantly, PD-L1 expression was not seen in neutrophils circulating in vessels close to the infected site, as observed by intravital microscopy. Instead, in human diffuse cutaneous leishmaniasis and in visceral leishmaniasis PD-L1-expressing cells have been detected in the blood circulation (12, 40). Similarly, higher levels of blood PD-L1^+^ neutrophils have been reported in tuberculosis and HIV-1-infected patients (7, 47), in patients with cancer (48), severe sepsis (49), while in rheumatoid disease, patients have neutrophils expressing PD-L1 in the synovial fluid, which can be related to the severity of the disease (50).

Another aspect identified in our study was the presence of PD-L1-expressing neutrophils in the draining lymph nodes of *L. amazonensis* infected mice. Upon analyzing the lymph nodes draining the chronic lesion we evidenced an increased number of neutrophils in this organ, and half of this population was expressing PD-L1. It has been reported that neutrophils have the ability to migrate to the lymph nodes, transporting and presenting antigens to T cells and inducing the activation and expansion of CD4^+^ T cells (51–54). We recently described in the draining lymph nodes of a non-healing *L. amazonensis* infection in BALB/c mice that both, CD4^+^ and CD8^+^ T cells expressed PD-1, and dendritic cells PD-L1 (28). Importantly, treatment of the *L. amazonensis-*infected mice with anti-PD-1 and anti-PD-L1 antibodies significantly increased IFN-γ production by T cells and decreased the parasite load (28). Our results in *Leishmania-*infected mice confirmed the presence of neutrophils in the lesion site in the first and second waves of neutrophil infiltration as well as in the chronic phase, and we perceived a relationship between PD-L1 expression and the cutaneous disease progression in the mouse.

It has been shown that neutrophil depletion with 1A8 monoclonal antibody enhanced T cell production of IFN-γ, suggesting a suppressor function of neutrophis (29). Here, we show the direct effect of *Leishmania* in the induction of PD-L1 on neutrophils, which interacting with CD8^+^ T cells, reduce IFN-γ production. Here, we demonstrated PD-L1 expression was detected in the skin of dogs severely infected with *L. infantum* (54), and in the spleen and in B cells of dogs infected with *L. infantum* (55–57). Several reports have identified the presence of neutrophils in chronic inflammatory infiltrate of patients with localized cutaneous (58, 59), diffuse cutaneous (60) and mucocutaneous leishmaniasis (61, 62).

Likewise to *L. amazonensis* induction of PD-L1 in human neutrophils, *L. braziliensis* infection in these cells shares the same capacity. Both promastigotes and amastigotes induced PD-L1 expression in neutrophils indicating an ability of this parasite to this phenotype. Finally, we analyzed lesions from patients with cutaneous leishmaniasis caused by *L. braziliensis*, evidencing an enhanced frequency of neutrophils expressing PD-L1 than in healthy controls. Similar increased PD-L1 transcripts have been reported in the lesions of patients infected with *L. braziliensis* (62). Our study demonstrated that neutrophils contributed to PD-L1 expression in the *Leishmania* lesion site.

Recently, it has been demonstrated by RNA-seq and immunohistochemistry that PD-1 and PD-L1 were highly up regulated in *L. braziliensis* skin lesion (63). Moreover, indicating an exhaustion process, anti-PD-L1/PD-L2 added to cultures of CD4^+^ and CD8^+^ T cells from cutaneous leishmaniasis patients increased the response to *L. braziliensis* antigen, and restored their IFN-γ response (63).

In summary, we show for the first time that *L. amazonensis* infection induces the expression of PD-L1 in neutrophils and that this expression increases with disease progression associated to chronic development of the disease. The induction of PD-L1 in neutrophils could favor the suppressive milieu that is important for the persistence of the parasite in human and experimental infections. Our finding is relevant because it opens new possibilities for therapeutic targets, and for understanding the local environment of the infection that may favor *Leishmania* growth.

## Conclusion

The present study demonstrated that both amastigotes and promastigotes of *L. amazonensis* and *L. braziliensis* are capable of inducing the expression of PD-L1 in murine and human neutrophils. It was also observed that, in a murine model, the expression is not present in the first wave of neutrophil infiltration, however, the expression increases in the lesion and in the draining lymph nodes as the disease progress. Importantly, we demonstrated the capacity of *Leishmania-*infected neutrophils to inhibit IFN-γ production by effector TCD8^+^ cells, which was reversed by anti-PD-L1. Overall, our findings strongly suggest that PD-L1 expressing neutrophils could participate in the modulation of the immune response, favoring *Leishmania* survival and persistence.

## Data Availability Statement

The raw data supporting the conclusions of this article will be made available by the authors, without undue reservation.

## Ethics Statement

The studies involving human participants were reviewed and approved by the HUCAM Ethical Committee and this study was registered under the number 735.274. The patients/participants provided their written informed consent to participate in this study. The animal study was reviewed and approved by Committee for Animal Use of the Universidade Federal do Rio de Janeiro (Permit Number: 161/18).

## Author Contributions

AF-M: Conceived and designed the analysis, data collection, data analysis and interpretation, scientific discussion, wrote and revised the manuscript. PL-G: Data collection. MA: Data collection, data analysis and interpretation. RG Data collection, data analysis, interpretation and critical revision of the article. LC: Data collection, data analysis, interpretation and critical revision of the article. GM: Scientific discussion. DG: Scientific discussion and critical revision of the article and funding. ES: Conceived and designed the analysis, scientific discussion, wrote and critical revised the manuscript and funding. HM: Conceived and designed the analysis, scientific discussion, IDEM acima and funding. All authors contributed to the article and approved the submitted version.

## Conflict of Interest

The authors declare that the research was conducted in the absence of any commercial or financial relationships that could be construed as a potential conflict of interest.

## Supplementary Material

Supplementary Figure 1 Comparison of PD-L1 expression in murine neutrophils from bone marrow and peritonium. Neutrophils recruited to the peritoneum with casein (Per) for 3 h or isolated from the bone marrow (BM) of mice. Cells were infected (1:10) with promastigotes (Pro) or amastigotes (Ama) of *L. amazonensis* for 4 h. Controls were performed with the same cells without infection (control). Data shown percentage of PD-L1+ cells are mean + SEM (N = 9-11). **p < 0.03, ***p < 0.0001.

Supplementary Figure 2 PD-L1 expression in human neutrophils infected with *L. amazonensis*. Human neutrophils (5x105) from healthy donors were incubated with CFSE-stained promastigotes (Pro) and amastigotes (Ama) of *L. amazonensis* (1:5 and 1:10), for 4 h. Control performed with neutrophils on medium. Cells were then analyzed by flow cytometry. Data shown as percentage of PD-L1 expression on human neutrophils, mean ± SEM (N= 5-6). ***p < 0.0001.

Supplementary Figure 3 Analysis of murine ear neutrophils after 18 h of infection. Cells were collected from *L. amazonensis*-infected ears after 18 h then submitted to flow cytometry. Controls were performed with uninfected mice (naïve). **(A)** Percentage of CD11b^+^ cells. **(B)** Number of CD11b^+^ cells. **(C)** Percentage of Ly6G^+^ CD11b^+^ cells. **(D)** Number of Ly6G^+^ CD11b^+^ cells. **(E)** Histogram of PD-L1 expression. Grey = Fluorescence minus one control (FMO) PD-L1, Blue = Naïve and Red = Infected. **(F)** Percentage of PD-L1^+^ Ly6G^+^ cells. **(G)** Number of PD-L1^+^ Ly6G^+^ cells, p=0.055. **(H)** MFI of PD-L1^+^ Ly6G^+^. Data are mean ± SEM from cells of individual mice (N = 4 mice/group).

Supplementary Figure 4 Dot plots of murine cells from lesions infected with *L. amazonensis*. Cells were collected from *L. amazonensis*-infected ears after 18 h. Controls were performed with uninfected mice (naïve) and FMO for **(A)** CD11b and **(B)** Ly6G (negative). **(A)** Dot plot of CD11b^+^ cells. **(B)** Dot plot of Ly6G^+^ CD11b^+^ cells. Data of individual mice (4 mice/group).

Supplementary Figure 5 Expression of PD-L1+ Ly6G+ cells in *L. amazonensis*-infected lesions. Cells were collected from *L. amazonensis*-infected ears after approximately 60 days. Controls were performed with uninfected mice (naïve). **(A)** Dot plot showing CD11b^+^ cells (PE-CD11b x SSC) on FMO for CD11b (negative), naïve and infected mice. **(B)** Percentage of CD11b^+^ cells. **(C)** Number of CD11b^+^ cells. **(D)** Dot plot showing PD-L1^+^ Ly6G^+^ and PD-L1^+^ Ly6G^-^ gates (PD-L1-APC x Ly6G-PerCP) on FMO Ly6G and PD-L1, naïve and infected mice. **p<0.008 Data are mean ± SEM of individual mice (4-5 mice/group) and are representative of three experiments producing the same result profile.

Supplementary Figure 6 Analyses of lesion neutrophils present a conspicuous expression of PD-L1. PD-L1 expression in neutrophils from healthy skin (HC, N = 8) and cutaneous leishmaniasis lesions (CL, N = 9). **(A)** Neutrophils are a percentage of nucleated cells. **(B)** Percentage PD-L1^+^ neutrophils. The P values were calculated using Student’s *t* test with Mann-Whitney test. ***p < 0.001.

Supplementary Figure 7 Gating strategy of CFSE+ neutrophils expressing PD-L1. **(A)** Neutrophils FSC x SSC. **(B)** Single cells - FSC-A x FSC-H. **(C)** CFSE^+^ and CFSE^-^ (CFSE x SSC). **(D)** Histogram of PD-L1 expression, Green = FMO (negative), Red= isotype, Orange = control not infected, Blue = infected.

Supplementary Figure 8 Gating strategy of neutrophils expressing PD-L1 on the mice ear and on draining lymph nodes. Ear cells after processing were analyzed as follows: **(A)** Cells FSC x SSC. **(B)** Single cells - FSC-A x FSC-H. **(C)** CD45^+^ (APC-cy7 x FSC-H). **(D)** CD11b^+^ and Ly6G^+^ (FITC x Percp). **(E)** Histogram of PD-L1 expression, Grey = FMO (negative), Black= naïve control (not infected), Red = infected. Draining lymph node cells were analyzed as follows: **(F)** Cells FSC x SSC. **(G)** Single cells - FSC-A x FSC-H. **(H)** CD11b^+^ (Pe-cy7 x SSC-A). **(I)** Ly6G^+^ CD11b^+^ (FITC x SSC-H). **(J)** Histogram of PD-L1 expression, Red = FMO (negative), Blue= naïve control (not infected), Orange = infected.

Supplementary Figure 9 Phenotype of CD8^+^ T cells differentiated in vitro. Spleen cells of C57BL/6 mice were processed, and CD8^+^ T cells purified by negative selection and sorted. **(A)** Pre-sort and Pos-sort SSC x CD8^+^, CD62L^+^ x CD44^+^. **(B)** On day 5, cells were stained with CD25^+^ x CD44^+^, CD127^+^ x CD44^+^ and CD62L^+^ x CD44^+^.

Supplementary Figure 10 Lesion development in *L. amazonensis*-infected mice. Mice were infected in the ear with *L. amazonensis* stationary-phase promastigotes (2x10^6^). Ear thickness was measured weekly using a thickness gauge.

Supplementary Figure 11 PD-L1-expressing neutrophils in infected ear lesions. BALB/c mice infected in the ear with *L. amazonensis* were injected with anti-PD-L1-APC and anti-Ly6G-FITC antibodies and imaged by intravital microscopy. Controls were performed with mice injected with PBS and non-infected (naïve) mice. Arrows show neutrophils expressing PD-L1. **(A)** Naïve mouse. **(B)** Control mouse injected with PBS. **(C)** Mouse infected for 1 h. **(D)** Mouse with chronic infection (60 days). Autofluorescence of the skin fur can be observed (Arrow head). Images were obtained using Nikon Eclipse Ti with an A1R confocal head equipped with four different lasers (excitation at: 405, 488, 546 and 647 nm) and emission bandpass filters at 450/50, 515/30, 584/50 and 663/738 nm. Objective Plan Apo λ 10x. Bars: 130 µm.

Movie S1 Neutrophils in the blood vessels in the infected ear did not express PD-L1. Mice infected in the ears with *L. amazonensis* for 1 h were injected with anti-PD-L1-APC and anti-Ly6G-FITC antibodies. White arrows indicate circulating neutrophils with no expression of PD-L1. Images were obtained using a Nikon Eclipse Ti with an A1R confocal head equipped with four different lasers (excitation at: 405, 488, 546 and 647 nm) and emission bandpass filters at 450/50, 515/30, 584/50 and 663/738 nm. Objective Plan Apo λ 10x. Presence of skin hair autofluorescence. Bar = 130 µm.

Movie S2 Migration of neutrophils without expression of PD-L1 to the infected ear. Mice infected in the ear with *L. amazonensis* for 1 h were injected with anti-PD-L1-APC and anti-Ly6G-FITC antibodies. Presence of skin hair autofluorescence (Arrow head). Images were obtained using a Nikon Eclipse Ti with an A1R confocal head equipped with four different lasers (excitation at four wavelengths: 405, 488, 546 and 647 nm) and emission bandpass filters at 450/50, 515/30, 584/50 and 663/738 nm. Objective Plan Apo λ 10x and 2x zoom. Bar = 62 µm.

Movie S3 Neutrophils expressing PD-L1 in a chronic *L. amazonensis* ear infection. Mice infected in the ear with *L. amazonensis* in the chronic infection (60 days) were injected with anti-PD-L1-APC and anti-Ly6G-FITC antibodies. Images were obtained using a Nikon Eclipse Ti with an A1R confocal head equipped with four different lasers (excitation at four wavelengths: 405, 488, 546 and 647 nm) and emission bandpass filters at 450/50, 515/30, 584/50 and 663/738 nm. Objective Plan Apo λ 10x. Bar =38,57 µm and 127,77 µm.

Movie S4 Circulating neutrophils not expressing PD-L1 in a chronic *L. amazonensis*. Mice infected in the ear with *L. amazonensis* in the chronic infection (60 days) were injected with anti-PD-L1-APC and anti-Ly6G-FITC antibodies. Presence of skin hair autofluorescence (Arrow head). White arrows indicate circulating neutrophils with no expression of PD-L1. Images were obtained using a Nikon Eclipse Ti with an A1R confocal head equipped with four different lasers (excitation at four wavelengths: 405, 488, 546 and 647 nm) and emission bandpass filters at 450/50, 515/30, 584/50 and 663/738 nm. Objective Plan Apo λ 10x. Bar = 130 µm.
