# Supplementary figures and images for: Leishmania Parasites Drive PD-L1 Expression in Mice and Human Neutrophils With Suppressor Capacity
Source: Front Immunol. 2021 Jun 15;12:598943. doi: 10.3389/fimmu.2021.598943 (PMC8240668; doi:10.3389/fimmu.2021.598943)

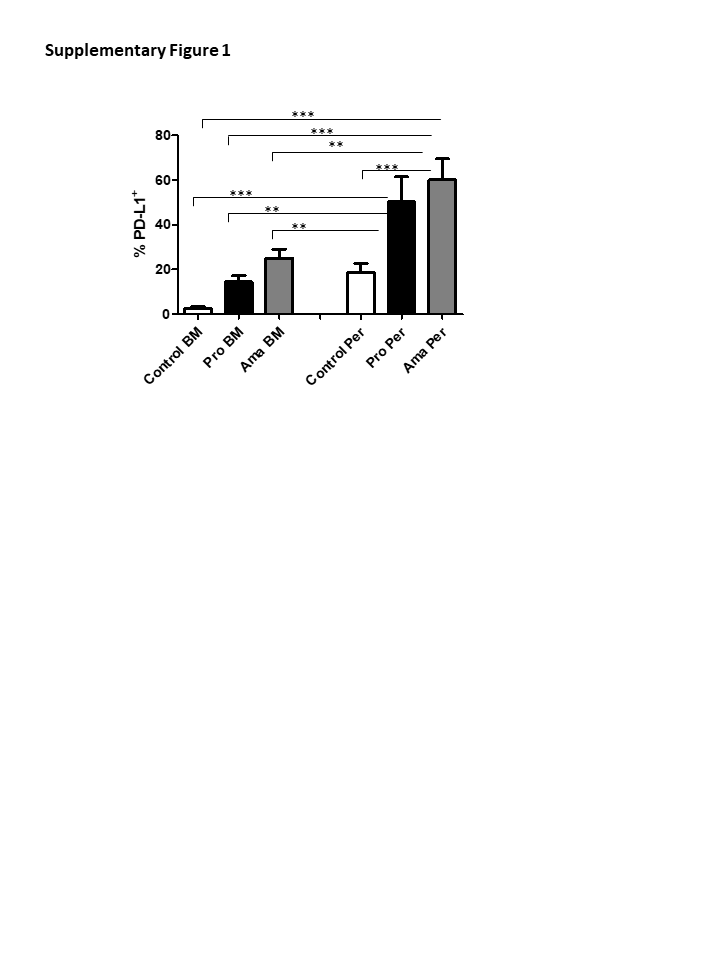

Supplement: Supplementary Figure 1 — Comparison of PD-L1 expression in murine neutrophils from bone marrow and peritonium. Neutrophils recruited to the peritoneum with casein (Per) for 3 h or isolated from the bone marrow (BM) of mice. Cells were infected (1:10) with promastigotes (Pro) or amastigotes (Ama) of L. amazonensis for 4 h. Controls were performed with the same cells without infection (control). Data shown percentage of PD-L1+ cells are mean + SEM (N = 9-11). **p < 0.03, ***p < 0.0001. [file Image_1.tif]

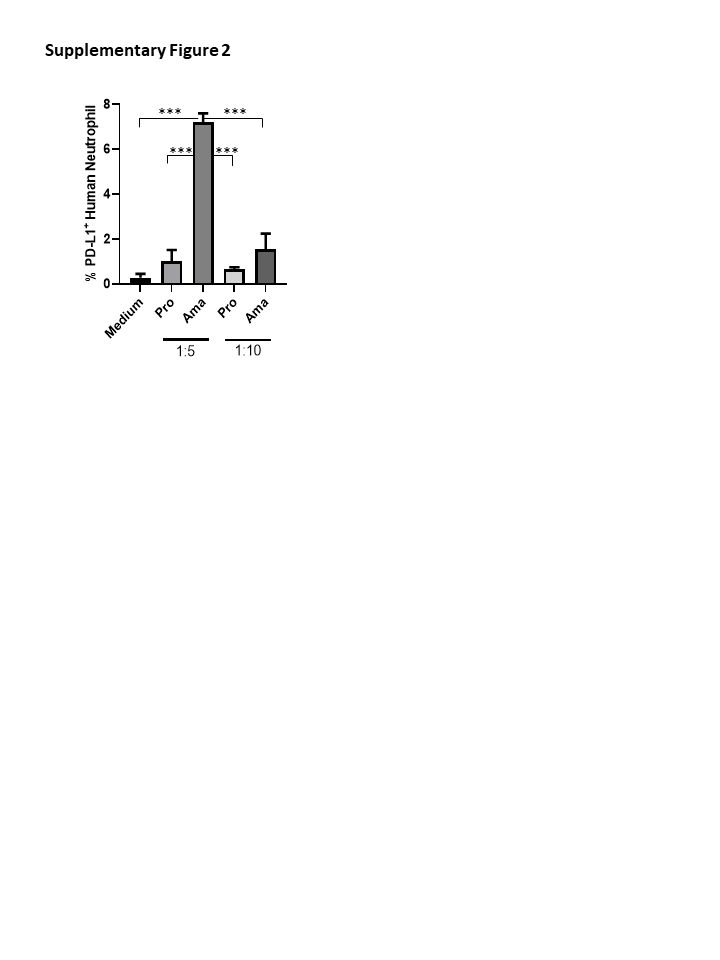

Supplement: Supplementary Figure 2 — PD-L1 expression in human neutrophils infected with L. amazonensis. Human neutrophils (5x10^5^) from healthy donors were incubated with CFSE-stained promastigotes (Pro) and amastigotes (Ama) of L. amazonensis (1:5 and 1:10), for 4 h. Control performed with neutrophils on medium. Cells were then analyzed by flow cytometry. Data shown as percentage of PD-L1 expression on human neutrophils, mean ± SEM (N= 5-6). ***p < 0.0001. [file Image_2.tif]

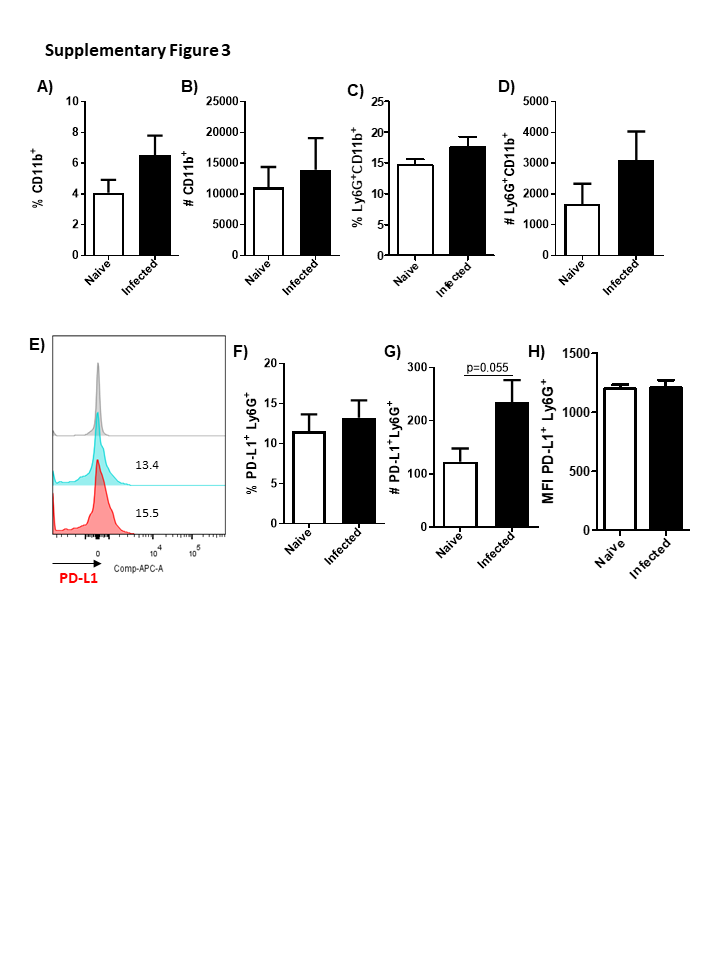

Supplement: Supplementary Figure 3 — Analysis of murine ear neutrophils after 18 h of infection. Cells were collected from L. amazonensis-infected ears after 18 h then submitted to flow cytometry. Controls were performed with uninfected mice (naïve). (A) Percentage of CD11b+ cells. (B) Number of CD11b+ cells. (C) Percentage of Ly6G+ CD11b+ cells. (D) Number of Ly6G+ CD11b+ cells. (E) Histogram of PD-L1 expression. Grey = Fluorescence minus one control (FMO) PD-L1, Blue = Naïve and Red = Infected. (F) Percentage of PD-L1+ Ly6G+ cells. (G) Number of PD-L1+ Ly6G+ cells, p=0.055. (H) MFI of PD-L1+ Ly6G+. Data are mean ± SEM from cells of individual mice (N = 4 mice/group). [file Image_3.tif]

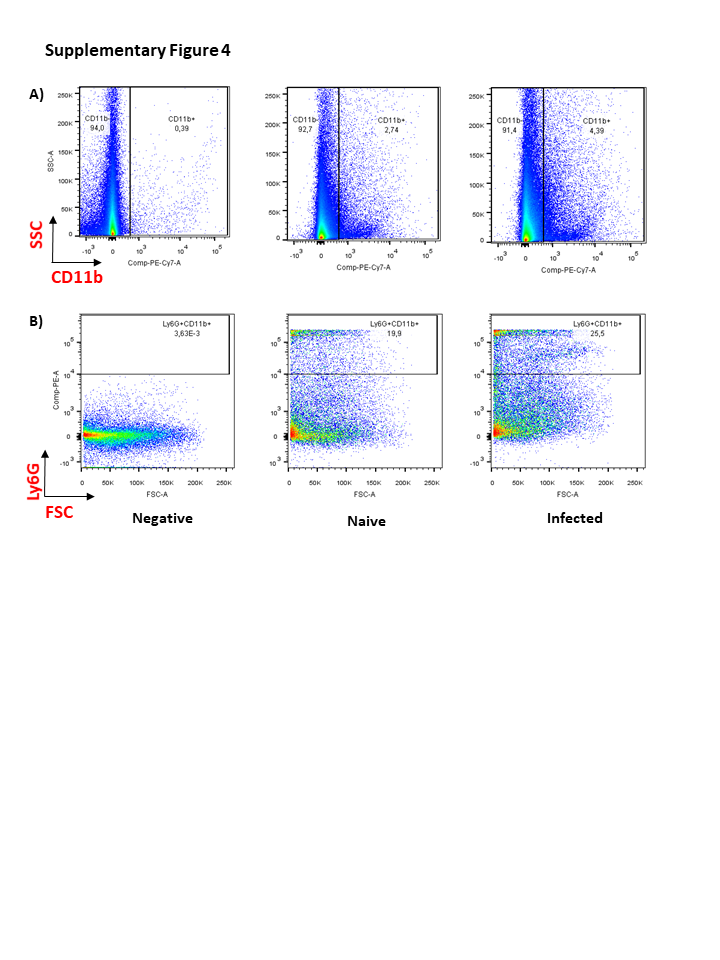

Supplement: Supplementary Figure 4 — Dot plots of murine cells from lesions infected with L. amazonensis. Cells were collected from L. amazonensis-infected ears after 18 h. Controls were performed with uninfected mice (naïve) and FMO for (A) CD11b and (B) Ly6G (negative). (A) Dot plot of CD11b+ cells. (B) Dot plot of Ly6G+ CD11b+ cells. Data of individual mice (4 mice/group). [file Image_4.tif]

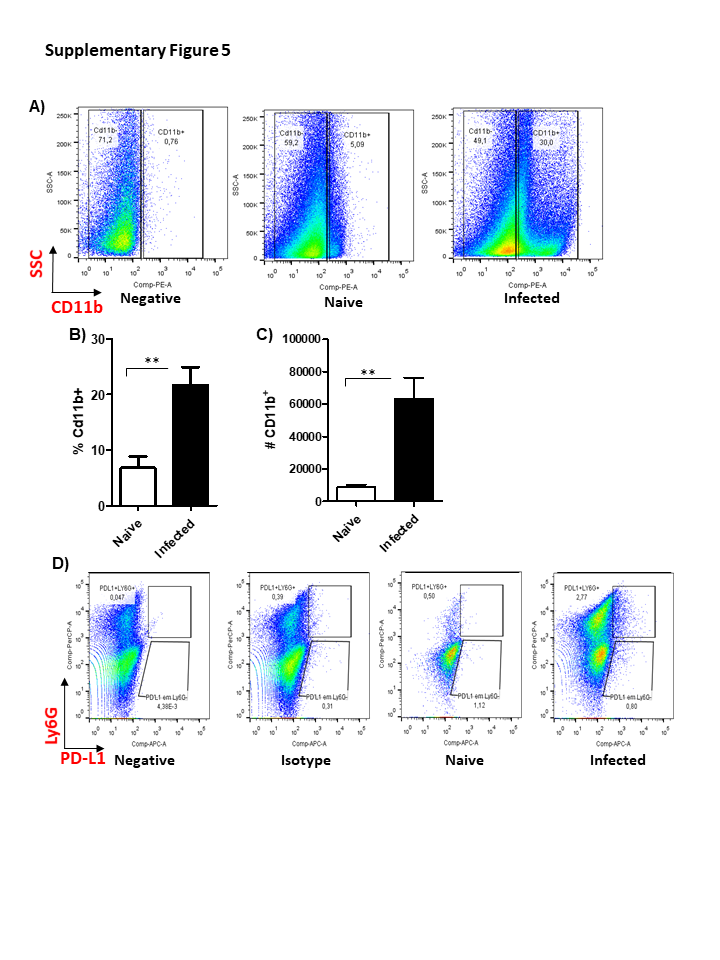

Supplement: Supplementary Figure 5 — Expression of PD-L1+ Ly6G+ cells in L. amazonensis-infected lesions. Cells were collected from L. amazonensis-infected ears after approximately 60 days. Controls were performed with uninfected mice (naïve). (A) Dot plot showing CD11b+ cells (PE-CD11b x SSC) on FMO for CD11b (negative), naïve and infected mice. (B) Percentage of CD11b+ cells. (C) Number of CD11b+ cells. (D) Dot plot showing PD-L1+ Ly6G+ and PD-L1+ Ly6G- gates (PD-L1-APC x Ly6G-PerCP) on FMO Ly6G and PD-L1, naïve and infected mice. **p<0.008 Data are mean ± SEM of individual mice (4-5 mice/group) and are representative of three experiments producing the same result profile. [file Image_5.tif]

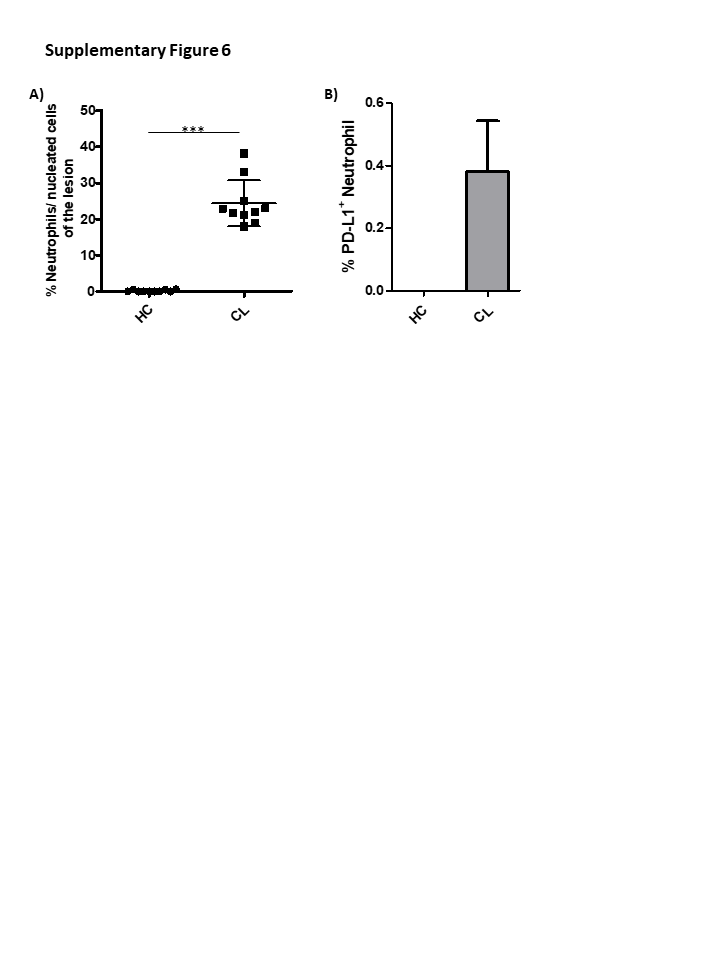

Supplement: Supplementary Figure 6 — Analyses of lesion neutrophils present a conspicuous expression of PD-L1. PD-L1 expression in neutrophils from healthy skin (HC, N = 8) and cutaneous leishmaniasis lesions (CL, N = 9). (A) Neutrophils are a percentage of nucleated cells. (B) Percentage PD-L1+ neutrophils. The P values were calculated using Student’s t test with Mann-Whitney test. ***p < 0.001. [file Image_6.tif]

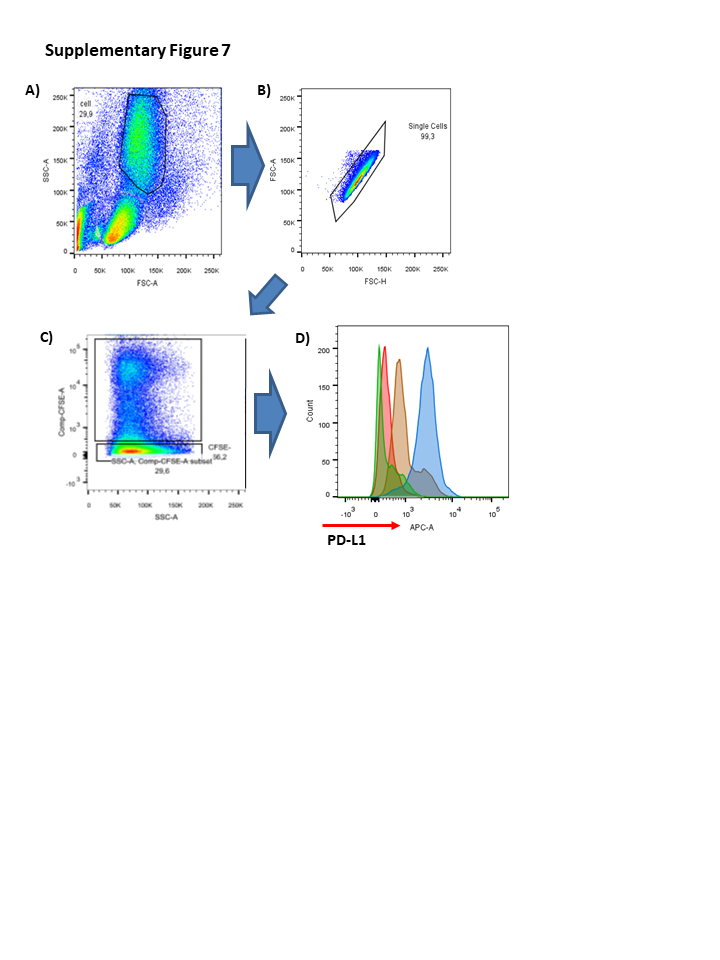

Supplement: Supplementary Figure 7 — Gating strategy of CFSE+ neutrophils expressing PD-L1. (A) Neutrophils FSC x SSC. (B) Single cells - FSC-A x FSC-H. (C) CFSE+ and CFSE- (CFSE x SSC). (D) Histogram of PD-L1 expression, Green = FMO (negative), Red= isotype, Orange = control not infected, Blue = infected. [file Image_7.tif]

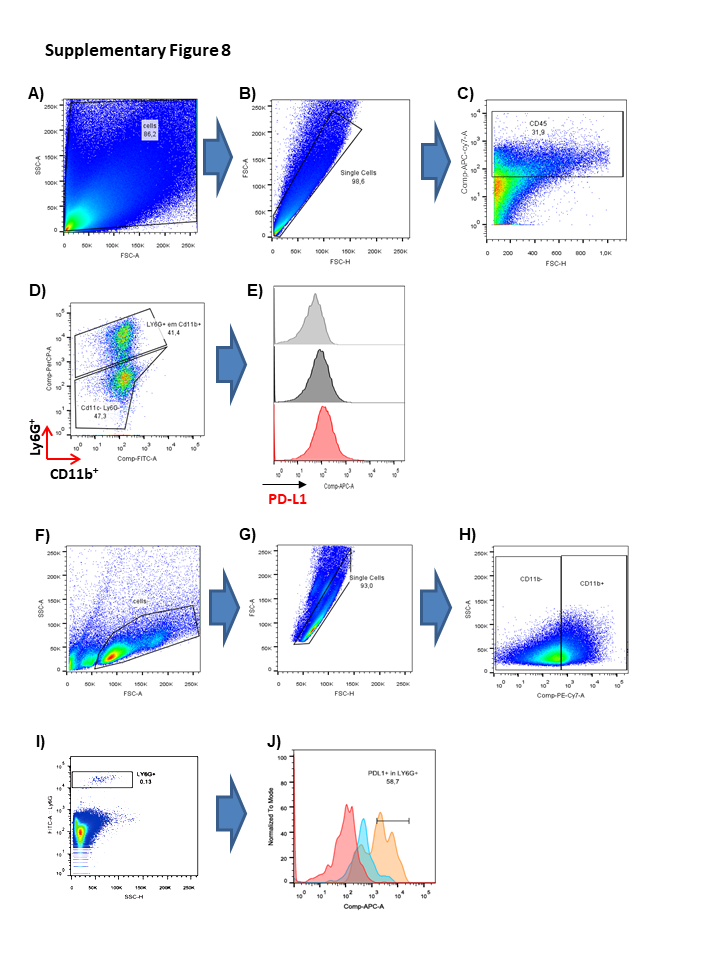

Supplement: Supplementary Figure 8 — Gating strategy of neutrophils expressing PD-L1 on the mice ear and on draining lymph nodes. Ear cells after processing were analyzed as follows: (A) Cells FSC x SSC. (B) Single cells - FSC-A x FSC-H. (C) CD45+ (APC-cy7 x FSC-H). (D) CD11b+ and Ly6G+ (FITC x Percp). (E) Histogram of PD-L1 expression, Grey = FMO (negative), Black= naïve control (not infected), Red = infected. Draining lymph node cells were analyzed as follows: (F) Cells FSC x SSC. (G) Single cells - FSC-A x FSC-H. (H) CD11b+ (Pe-cy7 x SSC-A). (I) Ly6G+ CD11b+ (FITC x SSC-H). (J) Histogram of PD-L1 expression, Red = FMO (negative), Blue= naïve control (not infected), Orange = infected. [file Image_8.tif]

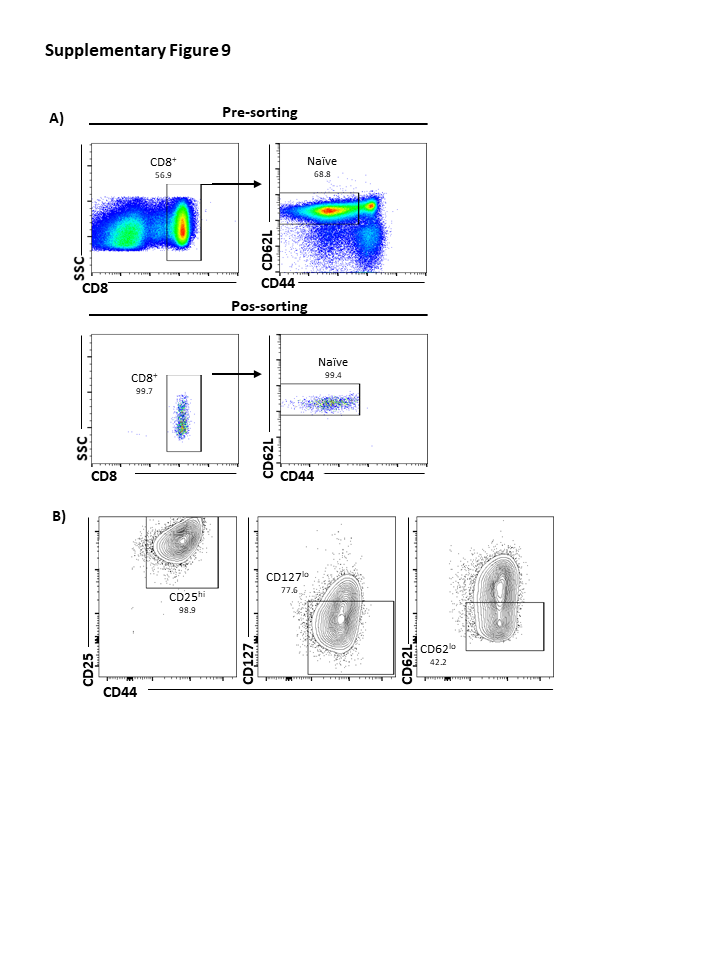

Supplement: Supplementary Figure 9 — Phenotype of CD8+ T cells differentiated in vitro. Spleen cells of C57BL/6 mice were processed, and CD8+ T cells purified by negative selection and sorted. (A) Pre-sort and Pos-sort SSC x CD8+, CD62L+ x CD44+. (B) On day 5, cells were stained with CD25+ x CD44+, CD127+ x CD44+ and CD62L+ x CD44+. [file Image_9.tif]

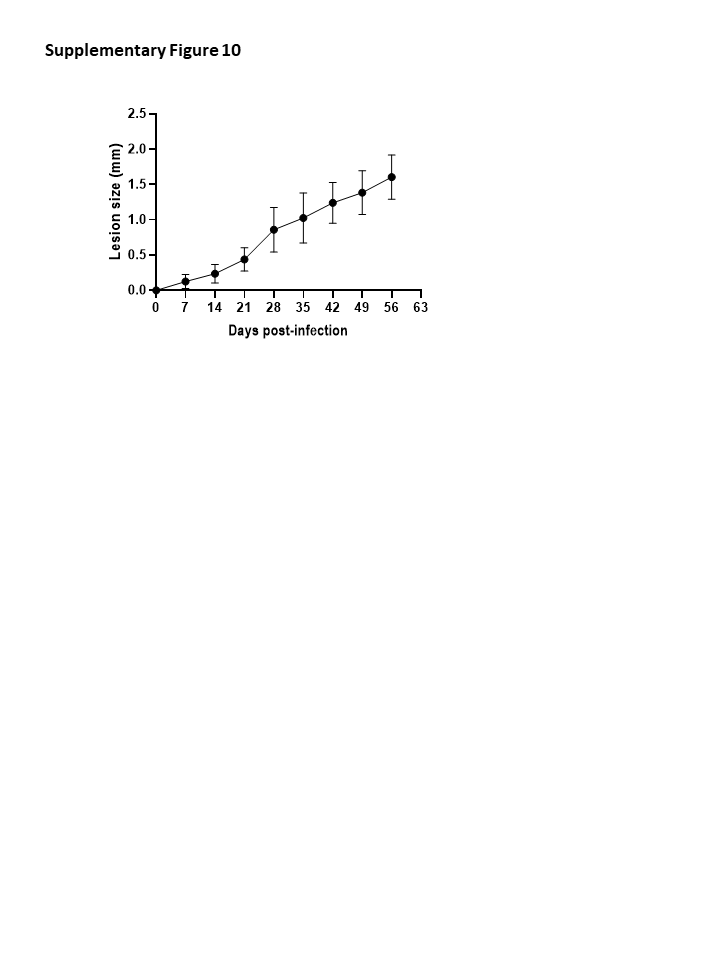

Supplement: Supplementary Figure 10 — Lesion development in L. amazonensis-infected mice. Mice were infected in the ear with L. amazonensis stationary-phase promastigotes (2x10^6^). Ear thickness was measured weekly using a thickness gauge. [file Image_10.tif]

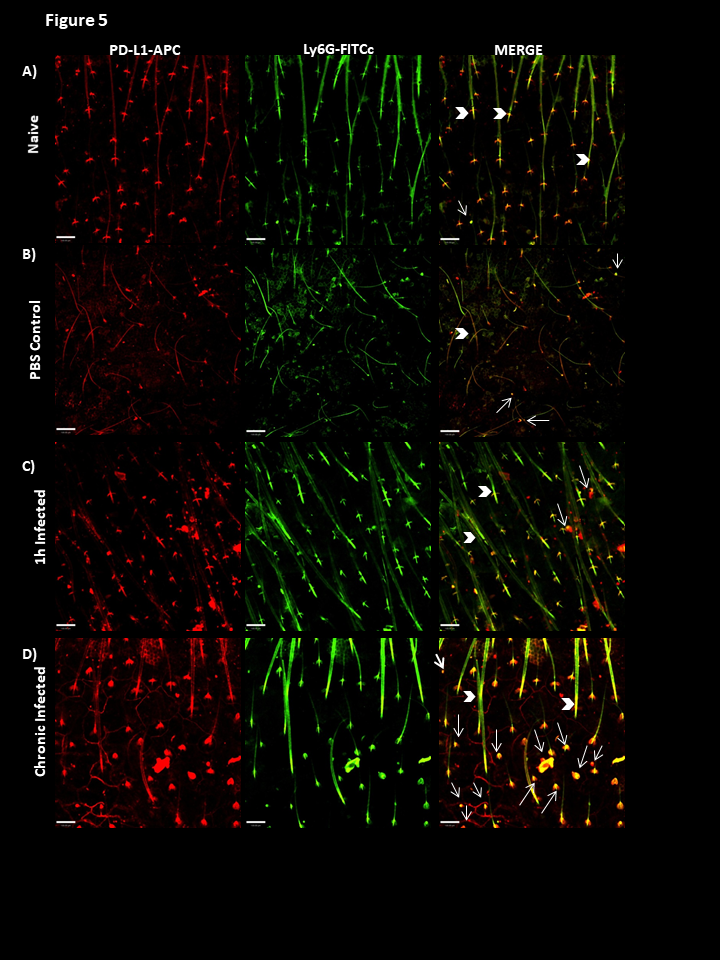

Supplement: Supplementary Figure 11 — PD-L1-expressing neutrophils in infected ear lesions. BALB/c mice infected in the ear with L. amazonensis were injected with anti-PD-L1-APC and anti-Ly6G-FITC antibodies and imaged by intravital microscopy. Controls were performed with mice injected with PBS and non-infected (naïve) mice. Arrows show neutrophils expressing PD-L1. (A) Naïve mouse. (B) Control mouse injected with PBS. (C) Mouse infected for 1 h. (D) Mouse with chronic infection (60 days). Autofluorescence of the skin fur can be observed (Arrow head). Images were obtained using Nikon Eclipse Ti with an A1R confocal head equipped with four different lasers (excitation at: 405, 488, 546 and 647 nm) and emission bandpass filters at 450/50, 515/30, 584/50 and 663/738 nm. Objective Plan Apo λ 10x. Bars: 130 µm. [file Image_11.tif]
